# Hemodialysis as an Effective Treatment for Combined Amlodipine and Metformin Overdose: A Case Report and Literature Review

**DOI:** 10.7759/cureus.40032

**Published:** 2023-06-06

**Authors:** Muhammad Samsoor Zarak, Sabah Khalafalla, Yashvardhan Batta, C Mere, Alem Mehari

**Affiliations:** 1 Internal Medicine, Howard University Hospital, Washington D.C., USA; 2 Cardiology, Howard University College of Medicine, Washington D.C., USA; 3 Nephrology, Howard University Hospital, Washington, D.C., USA; 4 Pulmonary and Critical Care, Howard University Hospital, Washington D.C., USA

**Keywords:** medical intensive care unit (micu), medication toxicity, urgent hemodialysis, metformin overdose, amlodipine posioning

## Abstract

The combined toxicity of amlodipine and metformin is a rarely reported phenomenon in the literature. The management varies depending on the clinical status of the patient. We present a case that was managed successfully with the early initiation of hemodialysis.

## Introduction

We present a 50-year-old patient with combined toxicity of amlodipine and metformin. We believe it is vital to report this case as there is limited literature on the management of the combined toxicity of prescribed medications. The early recovery of these patients depends on the modalities used to manage them. We report the utilization of emergency hemodialysis and its impact on the outcome of a patient with combined toxicity of amlodipine and metformin.

## Case presentation

A 50-year-old African American male with a past medical history of an alcohol use disorder, diabetes, hypertension, hyperlipidemia, obesity, and obstructive sleep apnea was transferred from an outside facility to our ICU for metformin and amlodipine overdose requiring medical management. He reported life stressors including family, living situation, and a recent altercation with his long-term partner, after which he attempted suicide by ingesting 20 tablets of metformin 500 mg and 20 tablets of amlodipine 10 mg. He smokes cigarettes daily with a 34-pack-year history and endorses binge drinking more than 14 drinks of gin per week. His last drink was one week before presentation to the hospital. The patient’s daily home medications include amlodipine 10 mg daily, ergocalciferol 1250 mcg weekly, latanoprost 0.005% drops, metformin 1000 mg twice a day, rosuvastatin 40 mg daily, telmisartan-hydrochlorothiazide 80 mg to 25 mg daily.

**Figure 1:**
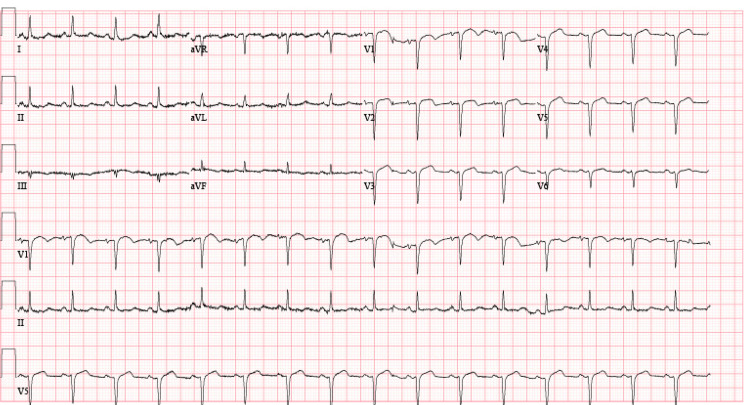
Electrocardiogram on presentation Normal sinus rhythm is observed with an anterolateral infarct of undetermined age

Prior to his transfer from the local medical center, he was given charcoal lavage, calcium gluconate, 5 L of normal saline fluids, 10 units of insulin with dextrose 5%, and ondansetron. The patient was alert and oriented on arrival at our ICU. He was tachycardic with a heart rate of 99 to 107 beats/minute, tachypneic at 25 breaths/minute, blood pressure of 112/54 mmHg, with a mean arterial pressure of 69 mmHg, and oxygen saturation of 96% on 3 L oxygen via nasal cannula. Arterial blood gas showed a pH of 7.538, partial pressure of carbon dioxide (PCO2) of 37.6, and partial pressure of oxygen (PO2) of 62.8. Electrocardiogram (EKG) showed normal sinus rhythm with anterolateral infarct of undetermined age (Figure 1).

**Figure 2:**
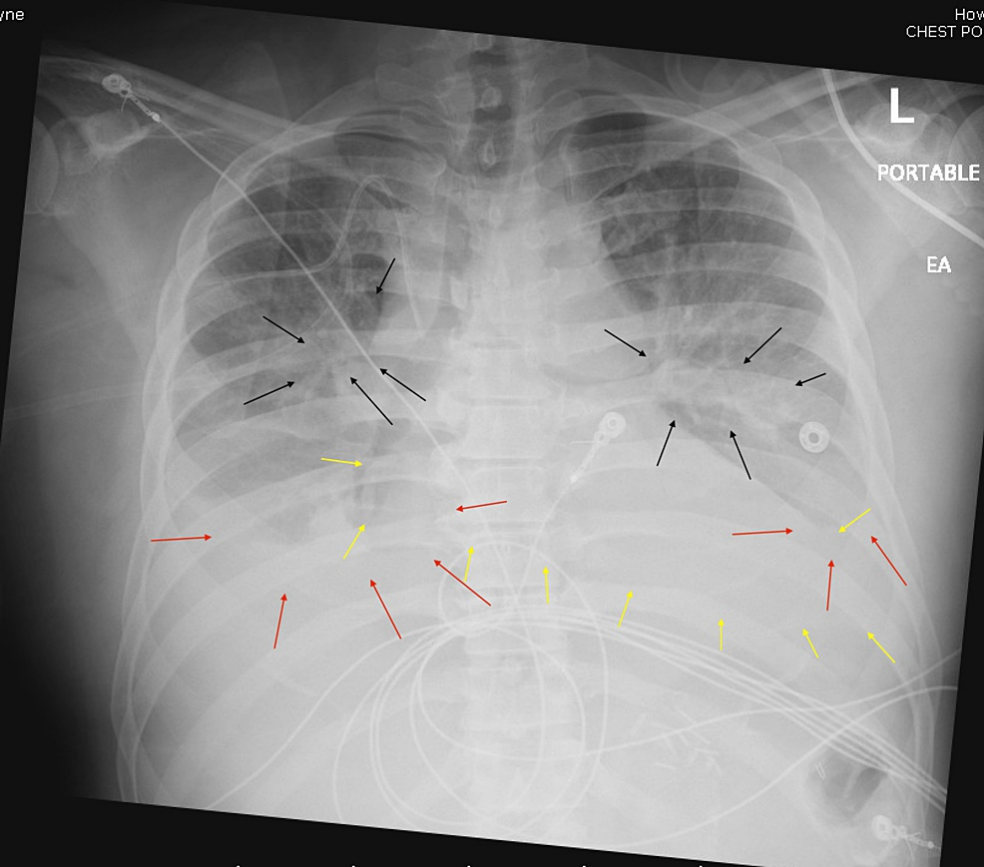
Chest X-ray on admission Black arrows: Pulmonary venous congestion, Red arrows: Bilateral pleural effusions, Yellow arrows: Enlarged cardiac silhouette

The chest X-ray showed pulmonary vascular congestion, bilateral pleural effusions, and an enlarged cardiac silhouette (Figure 2).

Labs showed serum bicarbonate of 12, anion gap of 24, lactic acidosis of 12.4 mmol/L, troponin of 0.052 ng/mL, and creatinine of 2.61 mg/dL (Table 1).

**Table 1:** Pertinent laboratory data during the course of hospitalization N/A: None available, HCO3: Bicarbonate

Variable (normal value)	Admission	12 hours	24 hours	36 hours	60 hours	84 hours	110 hours
Anion gap mEq/L (7-16)	24	20.4	12.9	11	11	13	12
HCO3 mEq/L (22-32)	12	21	30	34	32	28	26
Lactic acid mmol/L (0.5-2.2)	12.4	8.8	2.7	1.2	N/A	N/A	N/A
Troponin ng/mL (0.00-0.03)	0.052	0.090	0.211	0.135	N/A	N/A	N/A
Creatinine mg/dL (0.6-1.2)	2.61	3.24	2.93	2.32	1.48	1.33	1.06
Blood urea nitrogen mg/dL (7-25)	20	23	26	26	20	19	13
Glucose mg/dL (70-100)	133	287	195	146	126	132	123
Sodium mEq/L (135-145)	137	134	133	135	136	134	136
Chloride mEq/L (95-111)	101	93	90	93	96	97	102
Potassium mEq/L (3.5 -5.1)	4.0	3.6	3.1	3.1	3.4	3.6	3.9
Phosphorus mg/dL (2.5-4.5)	N/A	3.3	N/A	2.8	2.9	3.2	4.5
Magnesium mg/dL (1.7-2.5)	N/A	1.25	N/A	1.55	1.58	1.50	1.55
Glycosylated Hgb % (<= 5.7)	6.5	6.3	N/A	N/A	N/A	N/A	N/A
Calcium mg/dL (8.5-10.3)	9.5	8.2	8.1	8.8	8.5	8.6	9.1
Protein g/dL (6.3-8.3)	6.5	6.1	N/A	N/A	N/A	6.1	6.5
White blood cells K/uL (3.2-10.6)	16.83	9.73	N/A	9.66	8.77	7.46	6.50
Hemoglobin g/dL (14.6-17.8)	12	11.6	N/A	12.1	12.2	12.1	11.9
Hematocrit % (40.8-51.9)	36.6	34.1	N/A	35.1	35.7	35.3	35.0
Platelets K/uL (177-406)	248	178	N/A	142	157	199	230
Albumin g/dL (3.2-5.5)	4.06	3.74	N/A	N/A	N/A	3.44	3.59
Bilirubin mg/dL (0.2-1.2)	0.3	0.6	N/A	N/A	N/A	3.44	3.59
Alkaline phosphatase U/L (30-130)	54	49	N/A	N/A	N/A	52	55
Aspartate aminotransferase U/L (0-50)	32	25	N/A	N/A	N/A	17	28
Alanine aminotransferase U/L (0-55)	26	23	N/A	N/A	N/A	18	29

Subsequently, the patient became hypotensive and vasopressors was initiated. Norepinephrine was titrated up to 35 mcg/kg/min, vasopressin at 0.3 units/min and sodium bicarbonate drip was initiated at 125 ml/hr. Twenty-four hours post-presentation, patient developed chest pain, shortness of breath and oxygen saturation dropped to 80% requiring none invasive bilevel positive airway pressure ventilation (BiPAP) of 12/5 cmH2O with 50% of inspired oxygen. The Quinton catheter was placed for urgent hemodialysis in the setting of worsening anion gap metabolic acidosis and pulmonary edema. Cardiology was consulted in the context of chest pain and elevated troponin and patient was placed on acute coronary syndrome protocol. Cardiothoracic surgery team were also alerted just in case patient deteriorates and requires extracorporeal membrane oxygenation (ECMO).

After hemodialysis, follow up labs showed significant improvement. Serum bicarbonate improved from 12 to 30 mEq/L, the anion gap was corrected from 28 to 16 mEq/L, and lactic acid from 12.4 to 2.7 mm/L. However, troponin level was elevated initially from 0.052 to 0.178 ng/mL and trended down to 0.135 ng/ml. Similarly, serum creatinine initially trended up from 2.61 to 3.24 mg/dL then dropped to 2.32mg/dL. Fingerstick glucose was in the range of 133-287 which was managed to the goal of 180 with insulin low-dose sliding scale.

On the third day of ICU stay, the patient's clinical status improved. His BiPAP and oxygen need was decreased and transitioned to a nasal cannula. Vasopressors was weaned off. A psychiatry evaluation was obtained, and recommendations were followed which included peer recovery and education on substance use.

**Figure 3:**
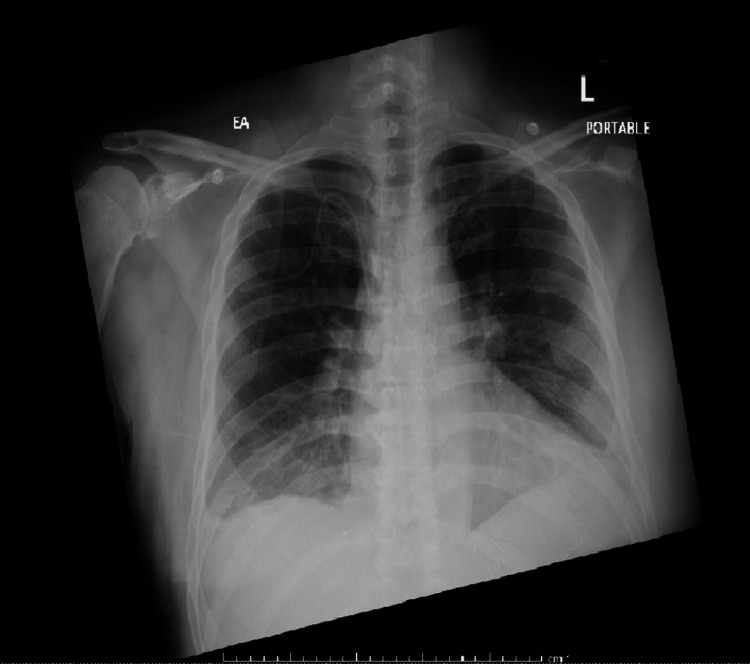
Chest X-ray on the fourth day of ICU stay showing significant improvement

On the fourth day of ICU care, the patient had normal hemodynamics. Metabolic profile significantly improved, and chest X-ray showed improvement in pulmonary edema (Figure 3).

**Figure 4:**
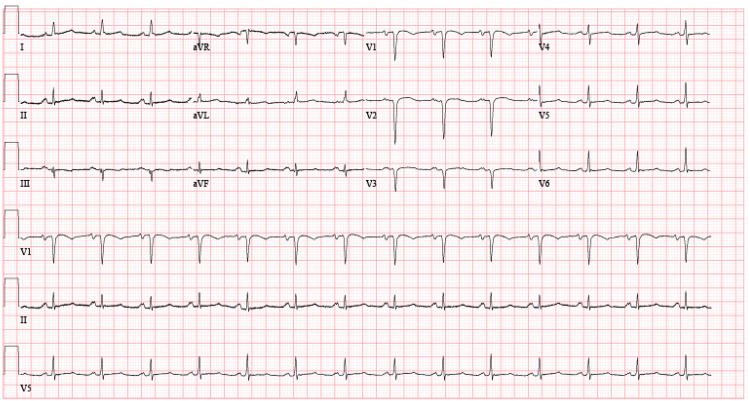
Electrocardiogram on day four of hospitalization

The IV maintenance fluid was held, and diuresis with intravenous furosemide was started. A repeat EKG (Figure 4) showed no changes from the previous EKG. Echocardiography reported preserved ejection fraction (EF) at 55% to 60%, without any wall motion abnormalities.

On the fifth day of ICU care, the patient continued doing well and denied any shortness of breath, chest pain, abdominal pain, nausea, or vomiting. The nuclear stress test showed no evidence of ischemia. Creatinine continued to downtrend to 1.33 mg/dL.

On the sixth day of hospitalization, his clinical status significantly improved with the normalization of kidney function, resolution of metabolic acidosis, and cardiogenic shock. The psychiatry team determined the patient was safe to discharge with close follow-up with his psychiatrist, therapist, and primary care provider.

## Discussion

Amlodipine is a dihydropyridine calcium channel blocker with the most prolonged half-life of its class at 30 to 50 hours. The FDA-approved indications include hypertension, chronic stable angina, vasospastic angina, and coronary artery disease (CAD) [1]. The mechanism of action involves inhibition of the voltage-gated L-type calcium channels, thereby lowering intracellular calcium, decreasing smooth muscle contraction, increasing smooth muscle relaxation, and vasodilation [2]. Side effects include refractory hypotension secondary to vasodilation, decreased chronotropy, tissue ischemia, subsequent lactic acidosis, and impaired pancreatic-islet insulin secretion. [3]

Metformin is a biguanide medication used to treat type 2 diabetes mellitus and the management of pre-diabetes [4]. It reduces blood glucose levels by decreasing gluconeogenesis, decreasing intestinal absorption, and increasing insulin sensitivity. Although generally safe and well-tolerated, side effects include diarrhea, nausea, vomiting, chest discomfort, headache, diaphoresis, weakness, rhinitis, hypoglycemia, and a black-boxed risk of lactic acidosis leading to metabolic acidosis [5].

A literature search on PubMed and google scholar showed three reported cases of combined toxicity of calcium channel blockers with metformin. Besides conservative management, the essential interventions used in the cases were the utilization of ECMO and continuous renal replacement therapy (CRRT) [6]. Another reported case used L-carnitine as a significant mode of management [7]. Among three reported cases, one succumbed [8]. In our case, early management with emergency dialysis addressed the metabolic acidosis earlier, which decreased the pressors requirement and gain early hemodynamic stability.

The discussion will be limited to the reported cases where patients survived. In the previously reported cases, the ingested doses of amlodipine were 300 mg and 400 mg (Table 2). In our case, it was 200 mg. The ingested units of metformin were 6.5 g and 20 g, while in our case, it was 20 . Besides pressors, the primary interventions used in case 1 was ECMO and CRRT; however, case 2 used L-carnitine as a modality to manage the patient.

**Table 2:** Summary of literature review ABG: Arterial blood gas, Ag: Anion gap, BP: Blood pressure, CCB: Calcium channel blockers, CRRT: Continuous renal replacement therapy, ECMO: Extracorporeal membrane oxygenation, HDI: High dose Insulin, HCO3: Bicarbonate, IHD: Intermittent hemodialysis; RR: Respiratory rate, Ca: Calcium, Na: Sodium, PCO2: Partial pressure of carbon dioxide, PO2: Partial pressure of oxygen

Authors	Details of Patients and Study	Toxicity	Labs	Pressor and Interventions	Status
Jeong et al. [6]	40-year-old male | BP: 70/50, Pulse: 60, RR: 22, Temperature: 36.5	CCBs: 400 mg, metformin: 20 g	Lactate: 127.32; HCO3: 9; ABG pH=7.206, PCO2=22.5, and PO2= 87.4	Levophed: 40 mcg/min, dopamine: 20 units, HDI: 110 units, lipid emulsion therapy: 1.5 ml/kg/min, Calcium gluconate: 2 g, NaHCO3: 100 ml/hr, ECMO, CRRT, IHD	Survived
St-Onge et al. [7]	68-year-old male | BP: 56/42, RR: 77	Amlodipine: 300 mg, metformin: 6.5 g	Lactic acid: 127; HCO3: 10; Ag: 22; ABG pH=7.0, PCO2=42	Ca gluconate: 3g, glucagon: 5 mg, levophed: 80 units, HDI: 80 units, lipid emulsion therapy: 120 ml of 20%, L-carnitine: 6g IV	Survived
Jović-Stosić et al. [8]	24-year-old female | BP: 80/40	Amlodipine: 150 mg, metformin: 10 g	Lactate: 44.14; ABGs pH=7.27, PCO2=28, and PO2=61	Gastric lavage dopamine: 40 units, glucagon: 15 mg, Ca gluconate: 20ml, lipid emulsion therapy: 20% 1000 ml	Expired

Patients with combined toxicity of amlodipine and metformin can be managed with urgent dialysis. Metformin leads to the production of lactic acidosis which causes metabolic acidosis. Urgent dialysis helps in the removal of metabolic acidosis from the body [9]. Metabolic acidosis impacts a patient's hemodynamics by lowering cardiac output and arterial blood pressure. Additionally, low pH also modulates the vascular tone [10,11]. The relaxation in the vascular tone by metabolic acidosis is mediated by nitric oxide i.e., (NO)/cyclic guanosine monophosphate (cGMP)-dependent, and prostacyclin (prostaglandin I2 (PGI2)/cyclic adenosine monophosphate (cAMP)-dependent) mediated pathways, which also leads to hyperpolarization of the cell membrane [12,13]. Therefore, early correction of metabolic acidosis decreased the pressors requirement in our patient. The metabolites of amlodipine remain in the body for a longer duration, which requires conservative management.

The significant secondary findings in our cases were elevated troponins. The previously reported cases had normal troponins levels or had not been reported in their cases. The amlodipine-mediated vasodilation explains the demand ischemia in our patients. Our patient received the acute coronary syndrome protocol; however, the echocardiogram and nuclear stress test did not show wall motion abnormalities or ischemic changes. Therefore, the elevated troponins were likely due to demand ischemia.

Furthermore, we did not utilize high-dose insulin and lipid emulsion therapy. Emergency hemodialysis and conventional management improved the hemodynamics in our patient. The need for vasopressors was also reduced on the third day of admission. The patient needed diuresis to manage the pulmonary edema. He improved clinically and was ultimately discharged on the seventh day of his admission.

## Conclusions

Introducing emergency dialysis will lead to significant outcomes in mortality and morbidity in patients with combined toxicity of amlodipine and metformin. Moreover, non-ST elevation myocardial infarction is also expected to be seen in such patients, which occurs as an outcome of amlodipine-mediated vasodilation. Therefore, there is a dire need for monitoring and managing cardiac complications as well. 
